# The Evelina Hospital

**Published:** 1902-05-17

**Authors:** 


### THE EVELINA HOSPITAL.

This hospital, which will be closed for about six months, in order that 24 new bedrooms for the nurses may be constructed, held its annual meeting on Wednesday, the 30th ult. We learn that there was a large attendance, but that some disappointment was caused by the non-arrival of the new president, Lord Ancaster, who had met with a slight accident. Unfortunately, the notice of the meeting did not reach us in time to enable our representative to attend, and we must therefore content ourselves with a review of the year's work.

The report opens with an allusion to the late Chairman, the Hon. Conrad Dillon, whose illness was announced at last year's annual meeting, and who has since passed away; his place has been filled by Mr. Charles Wightman, who joined the Committee in 1896.

### Patients.

The total number of in-patients treated during 1901 was 1,017. Of these, 775 were cured or relieved, 173 died, and 12 were discharged on other grounds. Fifty-seven remained on December 31st, but all have now left to enable the alterations to be carried out. The average time each child remained in the hospital was 22 days, and 61 beds, on an average, were occupied daily. There were 36,251 attendances of out-patients; 11,513 casualties and 6,677 new out-patients were treated. The number of daily average attendances was 115. The total number of operations performed was—in-patients, 416; out-patients, 1,693, 783 of which were performed under an anæsthetic. Of the 173 deaths recorded, the medical report states that 20 cases were admitted moribund and died within 24 hours, while 102 were under two years of age.

### Finance.

The total ordinary income in 1901 was £5,154. This included £291 13s. 4d. from the Hospital Sunday Fund, and £85 1s. from the Saturday Fund. Invested property realised £3,393, £66 being for rents. Out-patients' payments amounted to £92. Legacies were received to the amount of £60,449, of which £60,000 was the balance paid by the executors of Baron F. de Rothschild.

The ordinary expenditure was £6,821, and £834 was spent on repairs and building improvements; £60,000 was invested. There is £379 at the bankers to the credit of the building fund. The new rooms for the nurses will, we understand, cost something like £6,000.

